# Clinical, epidemiological, and genomic evidence on mpox in mainland China, 2022–2025: a scoping review

**DOI:** 10.3389/fmicb.2026.1880110

**Published:** 2026-08-24

**Authors:** Yanjie Peng, Yun Geng, Fan Yang, Ting Yang

**Affiliations:** 1 Taian Branch, Shandong Provincial Maternal and Child Health Care Hospital Affiliated to Qingdao University, Jinan, China; 2 Nutrition Division, Walvax Biotechnology Co., Ltd., Kunming, Yunnan, China; 3 Human Resources Department, The Third People’s Hospital of Kunming City, Infectious Disease Clinical Medical Center of Yunnan, Kunming, China

**Keywords:** mpox, monkeypox virus, mainland China, genomic epidemiology, lineage diversity, APOBEC‑like substitutions, scoping review

## Abstract

**Background:**

Since 2022, mpox has expanded globally with sustained human-to-human transmission and increasing evidence of MPXV genomic diversification. In mainland China, mpox evidence has accumulated rapidly, but clinical, epidemiological, and genomic findings remain fragmented.

**Methods:**

We conducted a scoping review of PubMed, CNKI, and WanFang databases up to March 2, 2026, and integrated literature-derived evidence with public MPXV sequences from GenBank, GenBase, and GISAID. Literature-derived data were used to map clinical-epidemiological characteristics, study-level genomic evidence, sequencing coverage, and reported lineage distribution. Curated public MPXV sequences were used for phylogenetic reconstruction, amino acid mutation profiling, and APOBEC-like substitution analysis.

**Results:**

Fifty-eight studies were included: 32 addressed clinical or epidemiological evidence only, 25 addressed genomic evidence only, and one contributed to both domains. Fourteen hospital-based studies summarized 951 cases, showing that reported cases were concentrated among young adult men, with frequent MSM exposure (798/897, 89.0%; 95% CI 86.7–90.9%) and HIV co-infection (469/951, 49.3%; 95% CI 46.1–52.5%). Public sequence curation identified 231 unique mainland China MPXV sequences, of which 230 were used for phylogenetic and mutation analyses. Literature-based genomic evidence comprised 26 genomic studies, 37 study-level genomic records, and 31 reported-case units, including 414 sequenced cases among 530 reported cases (78.1%; 95% CI 74.4–81.4%). Clade IIb predominated among sequenced cases (412/414, 99.5%; 95% CI 98.3–99.9%), with C.1.1 and C.1 most frequently represented. Within the available dataset, public genomes represented multiple lineages and were unevenly distributed across regions and time. Mutation analysis revealed dispersed amino acid variation and a predominance of G>A and C>T transitions (73.0%). After collapsing recurrent substitutions to unique genomic sites, the proportion of G>A/C>T transitions decreased to 35.8% and further to 17.8% under a strict APOBEC3 motif definition, indicating that the observed mutation spectrum includes both shared lineage-associated substitutions and sequence-context-based APOBEC-like patterns.

**Conclusion:**

Available evidence indicates multi-lineage MPXV circulation and in mainland China, but interpretation remains constrained by uneven sequencing and lack of individual-level clinical-genomic linkage. Integrated genomic surveillance and standardized data linkage are needed to better characterize MPXV transmission and evolution.

## 1 Background

Mpox is an infectious disease caused by monkeypox virus (MPXV), a double-stranded DNA virus of the Orthopoxvirus genus within the Poxviridae family (Mitjà et al., 2023). MPXV is currently classified into clade I, including Ia and Ib, and clade II, including IIa and IIb. These clades differ in geographic distribution, transmission patterns, and clinical severity: clade I has historically been associated with greater severity in Central Africa, whereas clade IIb was responsible for the large multinational outbreak that began in 2022 (Bunge et al., 2022; WHO, 2024).

The 2022 global outbreak marked a major shift in mpox epidemiology, with sustained human-to-human transmission across many non-endemic regions and a clinical profile frequently involving close physical or sexual contact networks (Dabie et al., 2023; Ghosh et al., 2023; Thornhill et al., 2022). At the same time, the outbreak generated unprecedented genomic data, revealing rapid lineage diversification, repeated international introductions, and a prominent accumulation of G>A and C>T substitutions consistent with APOBEC3-associated mutational signatures (Gigante et al., 2022; Molteni et al., 2023; Otieno et al., 2025). These developments have made genomic surveillance essential for resolving transmission pathways, tracking emerging lineages, and interpreting viral evolutionary patterns beyond what can be inferred from case counts alone.

Mainland China has experienced mpox in several overlapping phases. The first imported case was reported in Chongqing in September 2022, followed by the detection of locally acquired cases in 2023 and subsequent community transmission dominated by clade IIb viruses (National Health Commission of the People’s Republic of China, 2022; Ren et al., 2023). Recent regional genomic studies indicate that the available MPXV genomes from mainland China comprise several clade IIb lineages, including C.1, C.1.1, B.1.3, E.3, and E.4 (Cao et al., 2025; Li et al., 2025; Shi C. et al., 2025; Zhang et al., 2024; Zhao et al., 2026). Collectively, these regional reports are consistent with repeated introductions and heterogeneous local amplification, rather than a single lineage expansion. However, their regional scope and uneven sampling preclude a definitive reconstruction of nationwide lineage circulation. The more recent detection of clade I-related events, including Ib-associated infections, also underscores the need for continued monitoring of variant introduction and onward transmission risk (China CDC, 2025; Song et al., 2025; Sun et al., 2025).

Despite the increasing number of clinical, epidemiological, and genomic reports, the evidence base for mpox in mainland China remains fragmented. Clinical studies, surveillance summaries, and genomic investigations are often reported separately, and sequencing efforts are unevenly distributed across regions and time periods. Moreover, most available studies lack individual-level linkage between clinical characteristics and viral genomic data, limiting assessment of lineage-specific transmission, clinical manifestations, and outcomes.

These limitations preclude population-representative estimates of lineage distribution, definitive nationwide transmission reconstruction, and causal inference regarding associations between viral variation and clinical outcomes.

To synthesize this fragmented evidence base, we conducted a scoping review integrating literature-derived clinical and epidemiological evidence with publicly available MPXV genome sequences from mainland China. We aimed to map the available evidence and describe the lineage and mutation patterns represented in the curated public dataset during 2022–2025, while identifying gaps in genomic surveillance and clinical–genomic data linkage.

## 2 Methods

### 2.1 Study design and reporting framework

This scoping review mapped clinical, epidemiological, and genomic evidence on mpox in mainland China and integrated literature-derived findings with publicly available MPXV genome sequences. The review followed the Arksey and O’Malley framework and the Joanna Briggs Institute guidance for scoping reviews and was reported according to the PRISMA Extension for Scoping Reviews (PRISMA-ScR). The completed PRISMA-ScR checklist is provided in Supplementary Table S7.

### 2.2 Literature search strategy

PubMed, China National Knowledge Infrastructure (CNKI), and WanFang were searched on March 2, 2026, without lower publication-date restriction. PubMed was used for English-language records, and CNKI and WanFang were used for Chinese-language records. Search terms combined free-text and database-specific terms for mpox or monkeypox, China or mainland China, and clinical, epidemiological, surveillance, genomic, genotyping, or phylogenetic characteristics. The complete search strategies are provided in Supplementary Table S1.

### 2.3 Eligibility criteria and study selection

Eligible records were required to report laboratory-confirmed mpox cases in mainland China and to provide extractable clinical, epidemiological, outcome, genomic, genotyping, lineage, or phylogenetic information. Original research articles, surveillance reports, case series, and genomic reports were eligible. Single-case reports were retained only when they provided extractable genomic or epidemiological information relevant to evidence mapping (Tables 1–3).

**Table 1:** Demographic and exposure context of hospital-based mpox cases in mainland China.

Variable	Value	Contributing studies, *n*
Included studies	14	14
Cases included in analysis	951	14
Study period covered	2022–09 to 2024–12	14
Male sex	947/951 (99.6%; 98.9–99.8%)	14
Age, years^a^	Study-reported median/mean: 29.6–34.5; where ranges were available, ages spanned 19–51 years	14
MSM exposure	798/897 (89.0%;86.7–90.9%)	13
HIV co-infection^b^	469/951 (49.3%;46.1–52.5%)	14
Pre-existing HIV infection	207/432 (47.9%; 43.2–52.6%)	11
Co-infection with other STIs^c^	314/786 (39.9%; 36.6–43.4%)	11
Overseas travel history	18/490 (3.7%;2.3–5.7%)	7

Percentages were calculated using available denominators within hospital-based clinical series. Counts in this table should not be interpreted as deduplicated national totals. Two-sided Wilson 95% CIs are shown alongside the corresponding proportions.

^a^Age was summarized descriptively because reporting formats were heterogeneous across studies.

^b^For pre-existing HIV infection, one study with a non-comparable subgroup denominator was excluded from pooled calculation.

^c^Other sexually transmitted infections (STIs) were variably defined across studies.

**Table 2:** Clinical manifestations and outcomes of mpox cases based on study-level aggregated data.

Variable	Value	Contributing studies, *n*
Included studies^a^	32	32
Study period covered	2022–09 to 2024–12	32
Fever	1313/2049 (64.1%; 62.0–66.1%)	29
Any rash/skin lesion	2130/2194 (97.1%; 96.3–97.7%)	32
Anogenital or perianal lesions^b^	428/582 (73.5%; 69.8–77.0%)	8
Facial or head lesions^c^	507/1625 (31.2%; 29.0–33.5%)	20
Lymphadenopathy	747/2097 (35.6%; 33.6–37.7%)	28
Inguinal lymphadenopathy^d^	413/1078 (38.3%; 35.5–41.3%)	18
Pruritus	307/1019 (30.1%; 27.4–33.0%)	11
Pain	329/1134 (29.0%; 26.4–31.7%)	13
Asymptomatic infection	11/2199 (0.5%; 0.3–0.9%)	32
Hospitalized cases/proportion^e^	660/858 (76.9%; 74.0–79.6%)	15
Complications^f^	82/1006 (8.2%; 6.6–10.0%)	14
Death	13/1916 (0.7%; 0.4–1.2%)	29

Data were summarized at the study level using aggregated counts. Percentages were calculated using available denominators reported in each study. Because of potential overlap across studies, results should be interpreted as descriptive estimates rather than pooled patient-level measures. Two-sided Wilson 95% CIs are shown alongside the corresponding proportions.

^a^Included studies refer to studies with extractable study-level clinical or outcome data for Table 2.

^b^Only studies providing an extractable combined numerator for anogenital or perianal involvement were pooled; studies reporting genital and perianal lesions separately without an “any involvement” count were not combined for this row.

^c^This row includes studies reporting extractable facial/head involvement; studies reporting eye involvement only or combined trunk/head-face categories were handled conservatively according to extractable totals.

^d^Inguinal lymphadenopathy denominators were variable-specific in several studies; one study used lymphadenopathy-positive cases rather than the total cohort as denominator.

^e^Hospitalization data were highly heterogeneous and were often available only for subsets with treatment/outcome records; the pooled estimate should be interpreted with caution.

^f^“Complications” were variably defined across studies; in some reports this referred to specific complications rather than a standardized “any complication” outcome.

**Table 3:** Literature-based genomic evidence, sequencing coverage, and reported MPXV lineage distribution in mainland China.

Domain	Item	Value
Study coverage	Included studies reporting genomic evidence	26
	Literature genomic records	37
	Unique reported locations	25
	Earliest collection period reported	2022–09
	Latest collection date reported	2025-01-05
Reported sequencing activity	Total reported sequenced cases	414
	Total reported cases in studies with genomic evidence	530
	Sequenced cases among reported cases (%)	78.1% (74.4–81.4%)
	Reported-case units used for deduplicated counting	31
Reported clade profile	Reported clade IIb, *n* (%)	412 (99.5%; 98.3–99.9%)
	Reported clade Ib, *n* (%)	2 (0.5%; 0.1–1.7%)
Reported lineage profile	Reported lineage B.1, *n* (%)	3 (0.7%; 0.2–2.1%)
	Reported lineage B.1.3, *n* (%)	65 (15.7%; 12.5–19.5%)
	Reported lineage C.1, *n* (%)	142 (34.3%; 29.9–39.0%)
	Reported lineage C.1.1, *n* (%)	185 (44.7%; 40.0–49.5%)
	Reported lineage E.3, *n* (%)	17 (4.1%; 2.6–6.5%)
	Other reported lineages, *n* (%)	0 (0.0%; 0.0–0.9%)
Geographic distribution of reported sequenced cases	Top 3 location by sequenced cases	Beijing (*n* = 122), Shenzhen (*n* = 98) and Sichuan (*n* = 58)
	Sequenced cases contributed by top 3 locations, *n* (%)	278 (67.1%; 62.5–71.5%)

Reported cases refer to the total number of Mpox cases described in each study.

Sequenced cases represent the number of cases for which genome sequencing was performed, as reported in the original publications.

The proportion of sequenced cases among reported cases was calculated using deduplicated reported case counts.

Clade and lineage classifications were extracted as reported in the original studies, and lineage distribution percentages were calculated based on all records with available lineage-level annotation.

Two-sided Wilson 95% CIs are shown alongside the corresponding proportions and describe the included literature dataset rather than national sequencing coverage or lineage prevalence.

Records were excluded if they were reviews, commentaries, editorials, or other non-original articles; were not focused on mpox as the primary study population; reported cases outside mainland China; represented duplicate publications or overlapping datasets without additional extractable information; focused on special populations or atypical/severe presentations not suitable for descriptive evidence mapping; or lacked sufficient structured clinical or genomic data.

**Figure 1:**
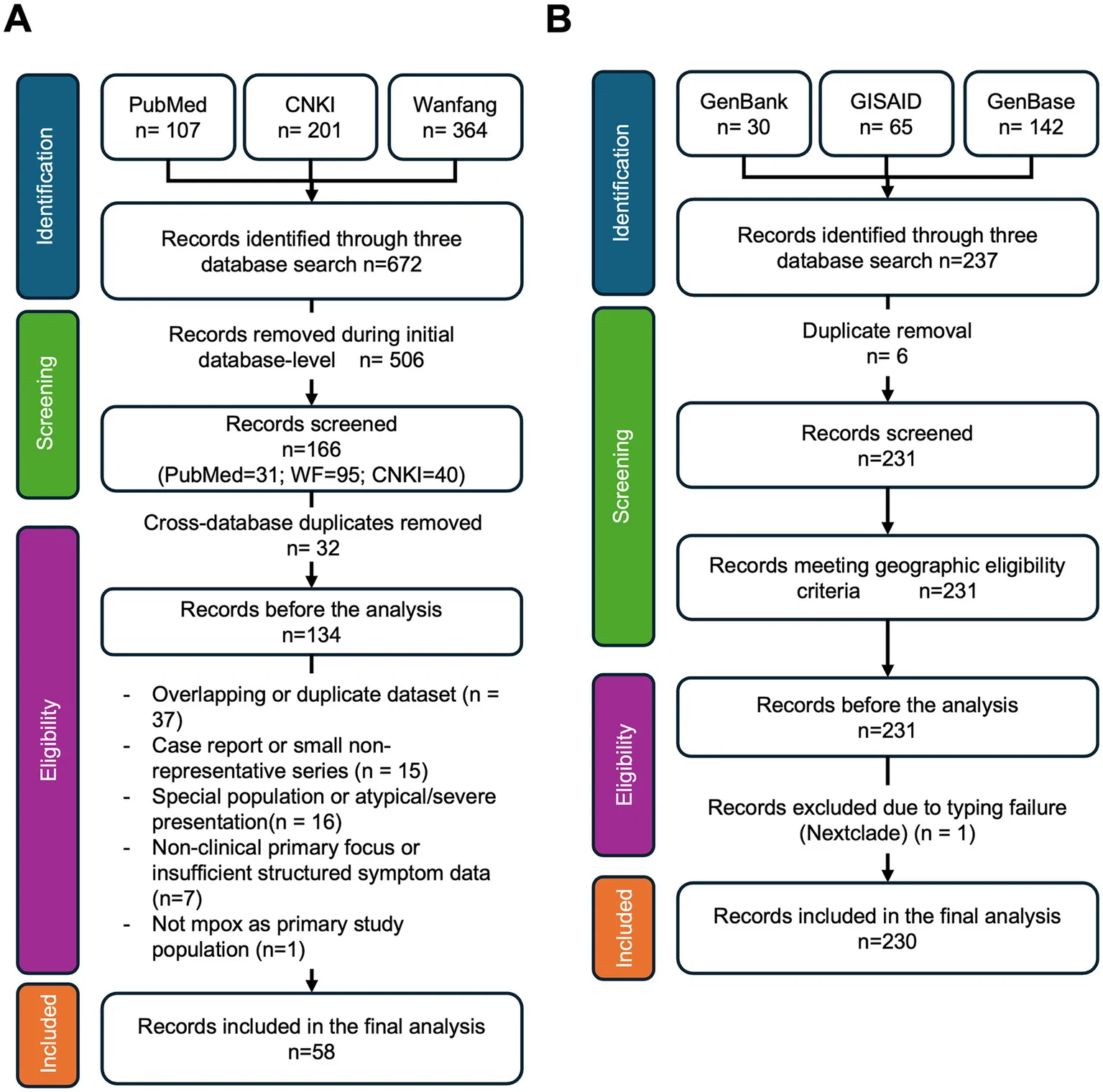
Integrated workflow for literature screening and MPXV genomic curation. PRISMA-ScR–based workflow showing the selection of literature records and genomic sequences included in this scoping review of mpox in mainland China. Literature records were identified from PubMed, CNKI, and WanFang, screened to establish a candidate pool, and assessed for eligibility **(A)**. Genomic sequences were retrieved from GenBank, GISAID, and GenBase, followed by duplicate removal and quality/typing assessment **(B)**. Exclusion reasons are shown at each stage. CNKI, China National Knowledge Infrastructure; PRISMA-ScR, Preferred Reporting Items for Systematic Reviews and Meta-Analyses extension for Scoping Reviews.

Records were managed in EndNote. Title/abstract screening and full-text eligibility assessment were performed by one reviewer and checked by a second reviewer; disagreements were resolved through discussion with other study members. The screening process and exclusion reasons are summarized in Figure 1 and Supplementary Table S2.

### 2.4 Data charting and evidence classification

A standardized data charting form was developed and iteratively refined. Data were extracted by one reviewer and checked by a second reviewer. Discrepancies were resolved by discussion with other study members. The extracted study characteristics and variables are provided in Supplementary Table S3.

For clinical and epidemiological evidence, extracted variables included study source, geographic location, study period, case number, demographic characteristics, exposure history, HIV etc. For literature-based genomic evidence, extracted variables included study location, collection period, reported cases, sequenced cases, sample source, clade, lineage etc.

Percentages were calculated using the number of cases with available information for each variable as the denominator. Unreported variables were not treated as negative findings. Because source populations may overlap across studies, aggregated clinical estimates were interpreted as descriptive study-level summaries rather than pooled patient-level measures.

### 2.5 Handling of overlap and reported-case units

Bibliographic duplicates were identified using title, authors, journal, year, DOI, and related publication metadata. For Chinese and English records or multiple reports describing the same population, records were compared by location, time period, authorship, case number, and reported variables. When datasets overlapped, the more comprehensive report, or the report with the broader time window and larger case number, was retained.

Clinical evidence was categorized by data source, including hospital-based clinical series, subnational surveillance or joint reports, and national surveillance summaries, because these sources differ in aggregation level and overlap risk. Counts from hospital-based studies were therefore not interpreted as deduplicated national totals. For genomic evidence, lineage-specific observations were retained as study-level genomic records, whereas records from the same publication were collapsed into a single reported-case unit when summarizing reported and sequenced case counts. In total, 37 lineage-specific genomic records corresponded to 31 reported-case units.

### 2.6 Public MPXV sequence retrieval and curation

Public MPXV genome sequences and metadata were retrieved from GenBank, GenBase, and GISAID up to March 2, 2026. Eligible records were human MPXV complete or near-complete genomes from mainland China with sufficient metadata for curation and downstream analysis. A total of 237 public sequence records were retrieved. Cross-database duplicates were identified using accession identifiers, strain names, collection dates, sampling locations, originating laboratories, and sequence length through semi-automated matching followed by manual review. After removal of six duplicate records, 231 unique mainland China sequences remained. One sequence, EPI_ISL_18354483, failed Nextclade lineage assignment and was excluded from phylogenetic and mutation analyses. Thus, 230 mainland China sequences were retained for sequence-based analyses. Sequence metadata are provided in Supplementary Table S4. This curated dataset represents publicly deposited sequences available at the time of retrieval and was not designed to constitute a population-representative sample of MPXV infections in mainland China.

### 2.7 Lineage assignment and phylogenetic analysis

Lineage assignment was performed using the Nextclade online platform. Multiple sequence alignment was conducted using MAFFT v7.526 with the auto option, allowing the program to select an appropriate alignment strategy according to dataset size and sequence characteristics. The phylogenetic dataset comprised 230 curated mainland China sequences and 38 reference sequences, resulting in 268 sequences for phylogenetic reconstruction. Metadata for the curated mainland China sequences and reference sequences are provided in Supplementary Table S4.

A maximum-likelihood phylogenetic tree was inferred using IQ-TREE v3.0.1. The best-fit nucleotide substitution model was selected using ModelFinder implemented in IQ-TREE, and branch support was assessed using ultrafast bootstrap approximation. The maximum-likelihood tree was used to visualize genetic relationships among the included public sequences and reference genomes. It was not used to reconstruct nationwide transmission chains, estimate lineage prevalence, or infer the number or direction of introduction events. The resulting tree was treated as an unrooted phylogeny and visualized in iTOL as a circular phylogram with branch lengths displayed. Temporal and spatial lineage summaries were generated in R.

### 2.8 Amino acid mutation analysis

Amino acid mutation analysis was performed using the 268-sequence phylogenetic dataset. Reference gene and protein annotations were based on GenBank record PP601195.1, selected for annotation completeness and suitability for coding-region mapping. Coding sequences were parsed from the GenBank flat file, translated, and compared with the corresponding reference protein sequences. Amino acid substitutions, deletions, and premature stop codons were summarized by gene/protein, lineage, and mutation type. Results are provided in Supplementary Table S5. Because lineage sample sizes were imbalanced and the aim was descriptive mutation profiling, no formal statistical comparison of amino acid mutation frequencies across lineages was performed.

### 2.9 APOBEC-like substitution analysis

APOBEC-like substitution patterns were analyzed using 230 mainland China sequences aligned to NC_063383.1. For each sequence, nucleotide substitutions were identified relative to the reference genome. Positions were retained only when both reference and query nucleotides were unambiguous A, T, C, or G bases; gaps, ambiguous bases, and non-substitution differences were excluded.

Broad APOBEC-like substitutions were defined as G>A and C>T transitions. Strict APOBEC3-associated motifs were defined by dinucleotide context as GA>AA for G>A substitutions preceded by A in the reference sequence and TC>TT for C>T substitutions followed by T in the reference sequence. Total substitutions were calculated by summing all substitution events across sequences. Unique genomic mutation sites were defined by collapsing recurrent substitutions at the same genomic position so that each position was counted once regardless of the number of sequences carrying the mutation.

The analysis generated descriptive summaries of the overall substitution spectrum, APOBEC-like transitions among all substitutions, APOBEC-like transitions after collapsing to unique genomic sites, strict APOBEC3-associated motifs, per-sequence APOBEC-like mutation burden, and lineage-level APOBEC-like substitution patterns. Results are provided in Supplementary Table S6. These analyses identify sequence-context-based APOBEC-like patterns and were not interpreted as direct experimental evidence of APOBEC editing. Lineage-level patterns were summarized descriptively; no formal statistical comparison between lineages or causal attribution to host-mediated editing was performed.

### 2.10 Data synthesis, visualization, and ethics

Data were synthesized descriptively, and no meta-analysis was performed. Clinical and epidemiological findings were summarized using available denominators for each reported variable. Two-sided 95% CIs for principal proportions were calculated using the Wilson score method without continuity correction. Given study-level aggregation, variable denominators, possible population overlap, and unavailable individual clinical–genomic linkage, no comparative, pooled, or association analyses were performed. CIs indicate conditional binomial precision, not meta-analytic or population-representative uncertainty; mutation-spectrum proportions were summarized descriptively. Literature-based genomic evidence was summarized at the study-record and reported-case-unit levels to describe sequencing coverage and lineage distribution. Sequence-based findings were interpreted descriptively and cross-referenced with literature-derived genomic evidence rather than treated as population-representative estimates. Analyses and visualizations were performed in R v4.6.0 using tidyverse, ggplot2, ggtree, ape, Biostrings, readxl, lubridate, svglite, scales, sf, and related packages. Geographic visualization used administrative boundary files from the National Geomatics Center of China.

As this review was designed to map available evidence rather than generate pooled effect estimates, formal risk-of-bias assessment was not performed. This study used published aggregate data and publicly available genomic sequences without individual-level identifiable information; therefore, ethical approval was not required.

## 3 Results

### 3.1 Evidence sources for clinical, epidemiological, and genomic mapping

Of 166 candidate records, 58 studies were included after deduplication and eligibility assessment (Figure 1; Supplementary Table S2). Of these, 32 contributed clinical or epidemiological evidence only (Chen et al., 2025; Duan et al., 2025; Guo et al., 2024; Hu et al., 2025; Huang et al., 2025; Ju et al., 2025; Liao et al., 2026; Lin et al., 2025; Liu and Wang, 2025; Liu H. et al., 2025; Liu K. et al., 2025; Liu et al., 2024; Liu Y. et al., 2025; Lu et al., 2025; Ma et al., 2025; Rao et al., 2025; Ren et al., 2024; Shao et al., 2024; Shi C. et al., 2025; Sun et al., 2024; Tan et al., 2026; Teng et al., 2025; Wang H. et al., 2025; Wang J. et al., 2025; Wang et al., 2024; Wei et al., 2025; Yang H. et al., 2024; Yang S. et al., 2024; Zhang L. et al., 2025; Zhao B. et al., 2024; Zhao et al., 2025; Zong et al., 2023), 25 contributed genomic evidences only (Bian et al., 2025; Cao et al., 2024; Cao et al., 2025; Han et al., 2024; He et al., 2025; Huang et al., 2024; Ji et al., 2024; Jiang et al., 2023; Li et al., 2025; Li et al., 2022; Lu et al., 2024; Pei et al., 2024; Shi C. et al., 2025; Song et al., 2025; Wan et al., 2023; Wu et al., 2024; Xu et al., 2025; Yang X. et al., 2024; Yu et al., 2023; Zhang et al., 2023; Zhang H. et al., 2025; Zhang et al., 2024; Zhao H. et al., 2024; Zhao K. et al., 2024; Zhao et al., 2023), and one contributed to both domains (Dou et al., 2023) (Supplementary Table S3). Separately, 231 unique public MPXV sequences were curated.

### 3.2 Epidemiological evidence landscape and geographic heterogeneity

**Figure 2:**
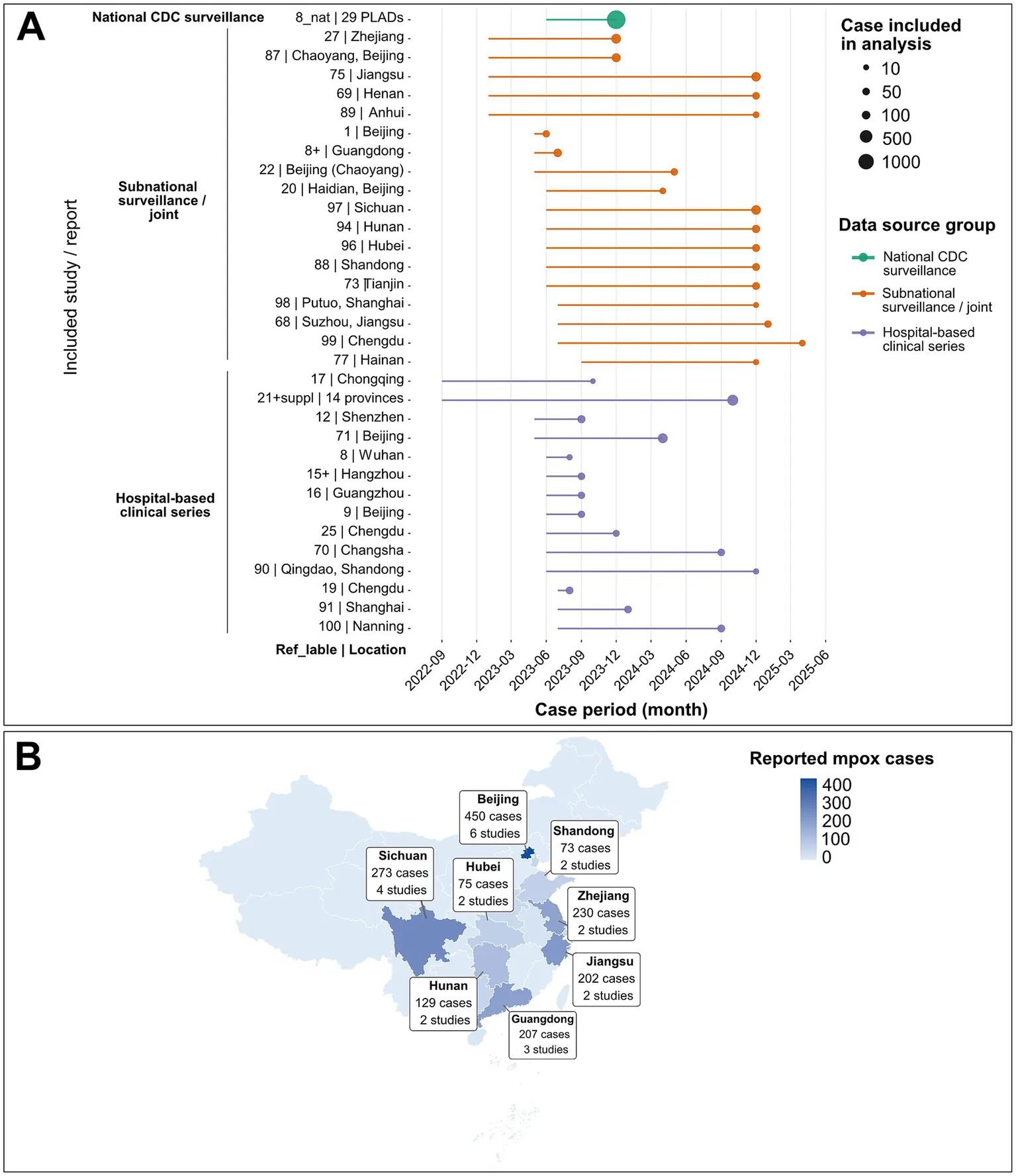
Epidemiological context and geographic heterogeneity of reported mpox evidence in mainland China. **(A)** Mpox cases across included records using a lollipop chart, stratified into hospital-based clinical series, subnational surveillance or joint reports, and national CDC surveillance. Each point represents a single study or report, and the horizontal axis indicates the number of reported cases. This panel highlights the heterogeneity in case counts across different data sources and reporting frameworks. **(B)** Geographic distribution of reported mpox cases across mainland China. Provinces are color-coded based on total reported case counts, and the number of included studies per province is annotated. The map was prepared using a standard map approved by the Ministry of Natural Resources of China (approval number: GS (2024) 0650). The map reveals both spatial clustering of reported cases and disparities in study representation across regions.

Epidemiological evidence comprised hospital-based clinical series (Guo et al., 2024; Hu et al., 2025; Liu and Wang, 2025; Liu et al., 2024; Liu Y. et al., 2025; Rao et al., 2025; Sun et al., 2024; Tan et al., 2026; Wang H. et al., 2025; Wang J. et al., 2025; Wang et al., 2024; Yang H. et al., 2024; Yang S. et al., 2024; Zhao et al., 2024), subnational surveillance or joint reports (Chen et al., 2025; Dou et al., 2023; Duan et al., 2025; Huang et al., 2025; Ju et al., 2025; Liao et al., 2026; Lin et al., 2025; Liu H. et al., 2025; Liu K. et al., 2025; Lu et al., 2025; Ma et al., 2025; Shao et al., 2024; Shi X. et al., 2025; Teng et al., 2025; Wei et al., 2025; Zhang L. et al., 2025; Zhao et al., 2025; Zong et al., 2023), and one national surveillance summary (Ren et al., 2024) (Figure 2A; Supplementary Table S3). Reported case counts and study representation were concentrated in Beijing, Zhejiang, Jiangsu, Guangdong, and Sichuan, whereas several other regions were sparsely represented (Figure 2B).

### 3.3 Demographic and exposure context of hospital-based mpox cases

Across 14 hospital-based studies (Guo et al., 2024; Hu et al., 2025; Liu and Wang, 2025; Liu et al., 2024; Liu Y. et al., 2025; Rao et al., 2025; Sun et al., 2024; Tan et al., 2026; Wang H. et al., 2025; Wang J. et al., 2025; Wang et al., 2024; Yang H. et al., 2024; Yang S. et al., 2024; Zhao et al., 2024) comprising 951 cases, patients were predominantly young adult men. MSM exposure was reported in 798/897 cases (89.0%; 95% CI, 86.7–90.9%), and HIV co-infection in 469/951 cases (49.3%; 95% CI, 46.1–52.5%). In a separate subset of cases with explicitly reported pre-existing HIV status, pre-existing HIV infection was reported in 207/432 cases (47.9%; 95% CI 43.2–52.6%), and co-infection with other sexually transmitted infections in 314/786 cases (39.9%; 95% CI 36.6–43.4%). Overseas travel history was uncommon (18/490, 3.7%; 95% CI 2.3–5.7%).

### 3.4 Study-level clinical manifestations and outcomes

At the study level, 32 studies (Chen et al., 2025; Dou et al., 2023; Duan et al., 2025; Guo et al., 2024; Hu et al., 2025; Huang et al., 2025; Ju et al., 2025; Liao et al., 2026; Lin et al., 2025; Liu and Wang, 2025; Liu H. et al., 2025; Liu K. et al., 2025; Liu et al., 2024; Liu Y. et al., 2025; Lu et al., 2025; Ma et al., 2025; Rao et al., 2025; Shao et al., 2024; Shi X. et al., 2025; Sun et al., 2024; Tan et al., 2026; Teng et al., 2025; Wang H. et al., 2025; Wang J. et al., 2025; Wang et al., 2024; Wei et al., 2025; Yang H. et al., 2024; Yang S. et al., 2024; Zhang L. et al., 2025; Zhao et al., 2024; Zhao et al., 2025; Zong et al., 2023) with extractable aggregated clinical manifestations or outcome data were included in this descriptive summary (Supplementary Table S3), whereas one national surveillance report was not pooled (Ren et al., 2024). Rash or skin lesions and fever were the predominant clinical manifestations, reported in 97.1% (2,130/2194; 95% CI 96.3–97.7%) and 64.1% (1,313/2049; 95% CI 62.0–66.1%) of cases with available data, respectively (Table 2). Anogenital involvement and lymphadenopathy were also frequently reported. Complications were uncommon and mortality was rare, whereas the relatively high reported hospitalization proportion varied substantially according to admission practices and study design.

### 3.5 Literature-based genomic evidence and sequencing coverage

The 26 genomic studies (Bian et al., 2025; Cao et al., 2024; Cao et al., 2025; Dou et al., 2023; Han et al., 2024; He et al., 2025; Huang et al., 2024; Ji et al., 2024; Jiang et al., 2023; Li et al., 2025; Li et al., 2022; Lu et al., 2024; Pei et al., 2024; Shi C. et al., 2025; Song et al., 2025; Wan et al., 2023; Wu et al., 2024; Xu et al., 2025; Yang X. et al., 2024; Yu et al., 2023; Zhang et al., 2023; Zhang H. et al., 2025; Zhang et al., 2024; Zhao et al., 2024; Zhao et al., 2024; Zhao et al., 2023) yielded 37 study-level genomic records from 25 locations, which were collapsed into 31 reported-case units for deduplicated case counting (Table 3; Supplementary Table S3). Sequencing was reported for 414 of 530 cases in these studies; this literature-based proportion does not represent national sequencing coverage.

**Figure 3:**
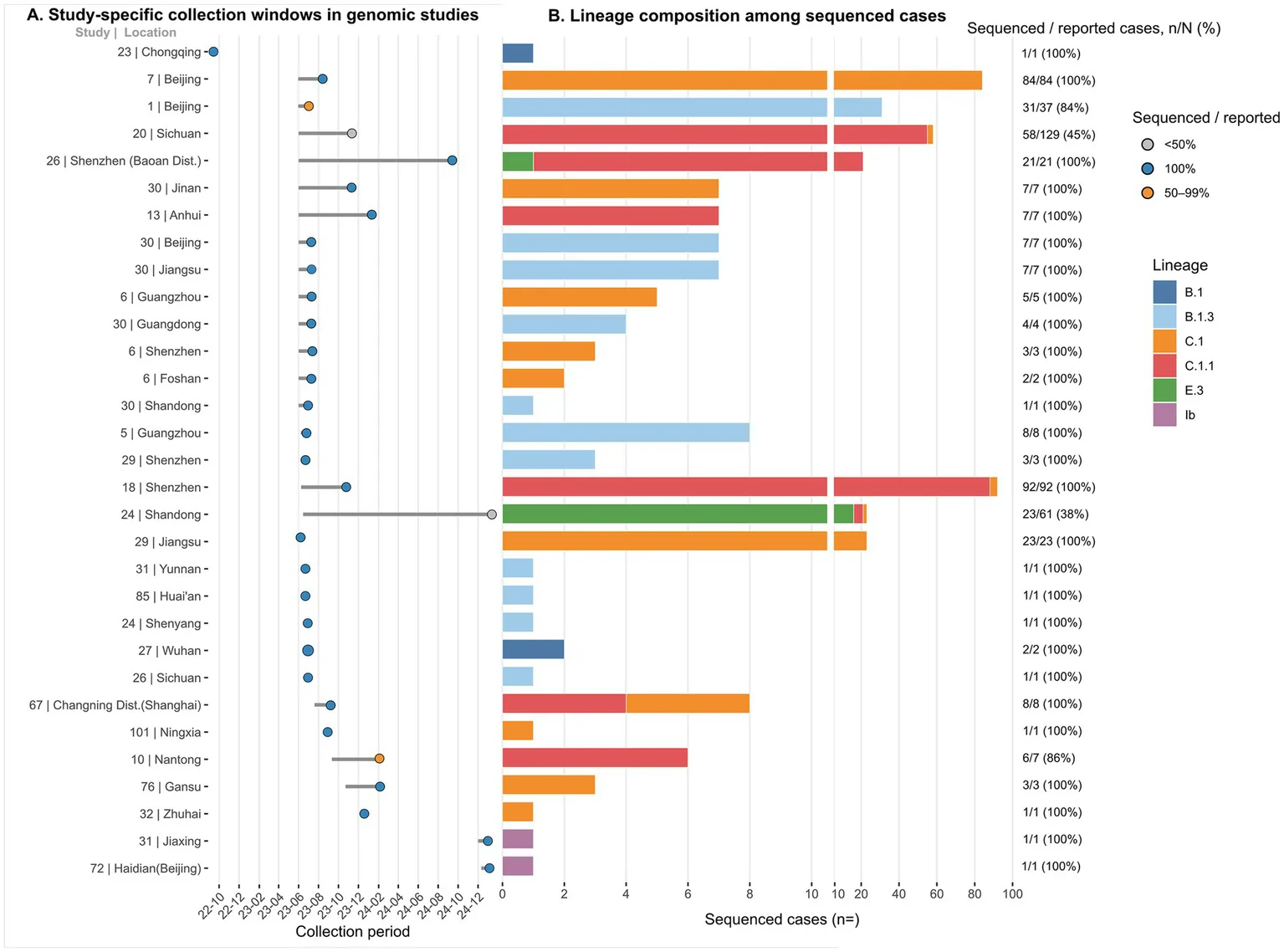
Study-level genomic surveillance coverage and lineage composition of mpox outbreaks with genomic evidence in Mainland China. **(A)** Study-specific collection windows and sequencing coverage. Each row represents one included study or report with extractable genomic information. Horizontal lines indicate the reported collection period. Fixed-size endpoint symbols indicate the sequencing-coverage category (<50%, 50–99%, or 100%), and the right-hand column reports the exact number of sequenced and reported cases for each study. **(B)** Lineage composition among sequenced cases in each study. Stacked bars show the number of sequenced cases assigned to each lineage. The x-axis is displayed with a scale break (0–10 and 10–100) to allow visualization of both small and larger sequencing datasets.

Sequenced cases were concentrated in Beijing (Dou et al., 2023; Wu et al., 2024; Zhang et al., 2023), Shenzhen (Shi C. et al., 2025; Wan et al., 2023; Yu et al., 2023; Zhang et al., 2024), and Sichuan (Cao et al., 2024; Cao et al., 2025), which together contributed 67.1% (95% CI 62.5–71.5%) of the literature-derived sequences. C.1.1 (185/414, 44.7%; 95% CI 40.0–49.5%) was the most frequently represented lineage, followed by C.1 and B.1.3, although reporting windows and lineage composition varied substantially across studies (Figure 3; Table 3).

### 3.6 MPXV phylogeny, lineage diversity, and amino acid mutation profiles

**Figure 4:**
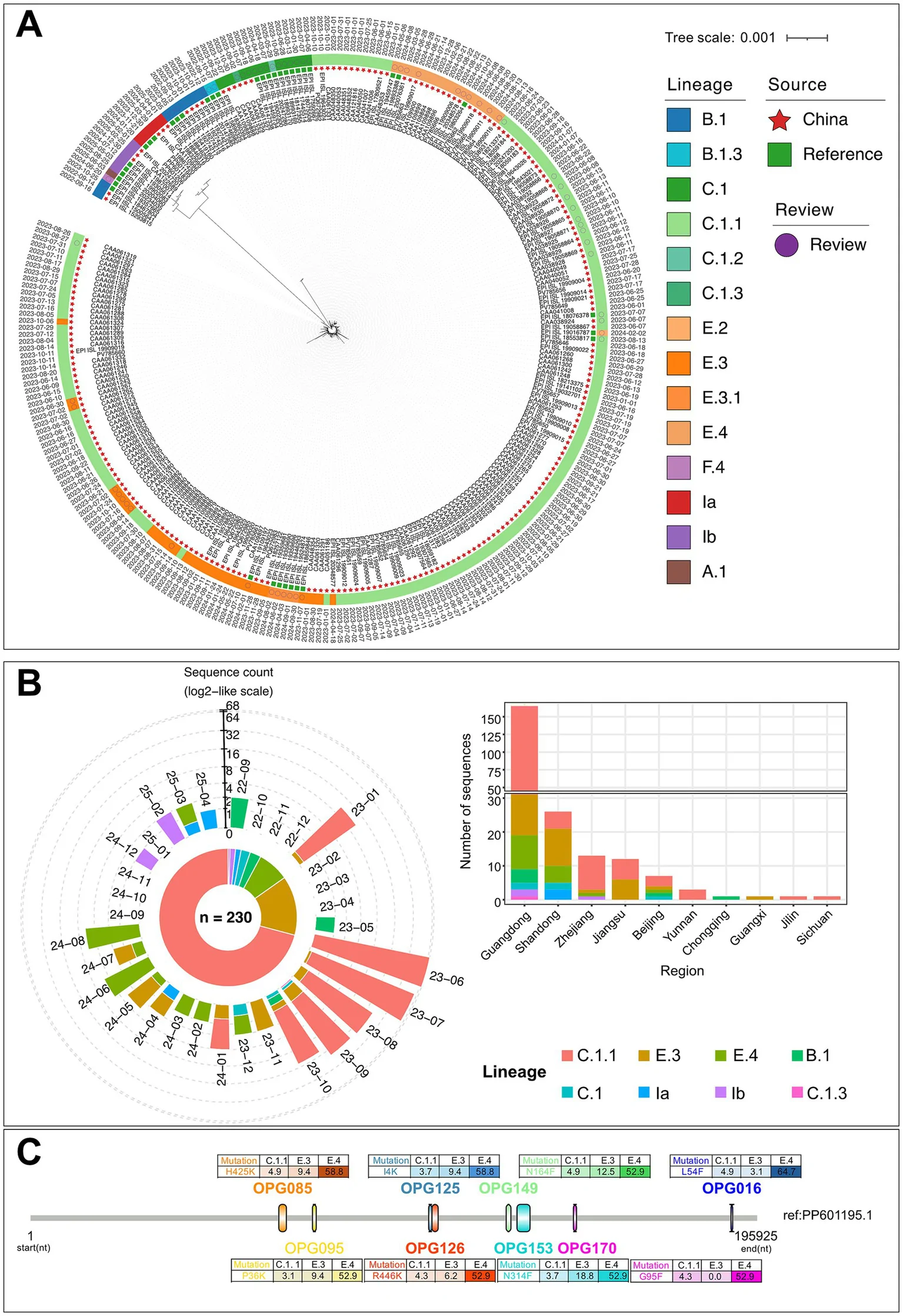
Genomic epidemiology, lineage diversity, and amino acid mutation profiles of MPXV sequences from Mainland China. **(A)** Maximum-likelihood phylogenetic tree constructed from 268 sequences, including 230 curated Mainland China sequences and 38 reference sequences. Lineage information was assigned using Nextclade and shown alongside the phylogenetic clustering pattern. The tree demonstrates that the distribution of Mainland China sequences across multiple MPXV lineages and phylogenetic clusters. **(B)** Left: Temporal distribution of major lineages based on available sample collection dates from September 2022 to April 2025. Right: Geographic distribution of lineages across provinces based on available geographic metadata. **(C)** Amino acid mutations were identified from genomic sequences and annotated relative to the reference genome. Mutations were mapped to corresponding viral proteins to characterize their distribution across the viral proteome.

The unrooted phylogeny placed the curated mainland China sequences across multiple lineages, including B.1, B.1.3, C.1, C.1.1, E.3, E.4, Ia, and Ib (Figure 4A; Supplementary Table S4), consistent with lineage diversity in the currently available public dataset.

Temporal and geographic summaries showed uneven sequence representation across time and regions (Figure 4B). Sequence availability was concentrated in 2023, particularly during mid-2023, with fewer sequences reported in later periods. Based on the available collection dates in Supplementary Table S4, the public genomes included in this analysis covered samples collected from 14 September 2022 to 1 April 2025. Regionally, Guangdong contributed the largest number of submitted sequences, followed by Shandong, Zhejiang, Jiangsu, and Beijing. Lineage composition varied across regions, with C.1.1 contributing a substantial proportion of sequences in multiple provinces, while other lineages appeared at lower frequencies or in specific locations. Therefore, the predominance of C.1.1 among public genomes should be interpreted in the context of the temporal and geographic composition of available sequences, rather than as a uniform estimate of lineage prevalence across all regions and time periods.

Amino acid mutation analysis was conducted to characterize lineage-associated variation across viral proteins (Figure 4C; Supplementary Table S5), using the curated public sequence dataset described above. Several mutations showed higher frequencies in E.4 compared with C.1.1 and E.3 within the available sequence dataset, including H425K, L54F, I4K, R446K, P36K, N164F, N314F, and G95F. These mutations were distributed across multiple viral proteins rather than concentrated within a single genomic region. These findings indicate that lineage-associated amino acid variation is dispersed across the viral proteome. Formal statistical comparison of mutation frequencies between lineages was not performed due to imbalanced lineage sample sizes and the presence of lineage-specific mutations in relatively small subsets.

### 3.7 APOBEC-like substitution patterns across MPXV lineages

**Figure 5:**
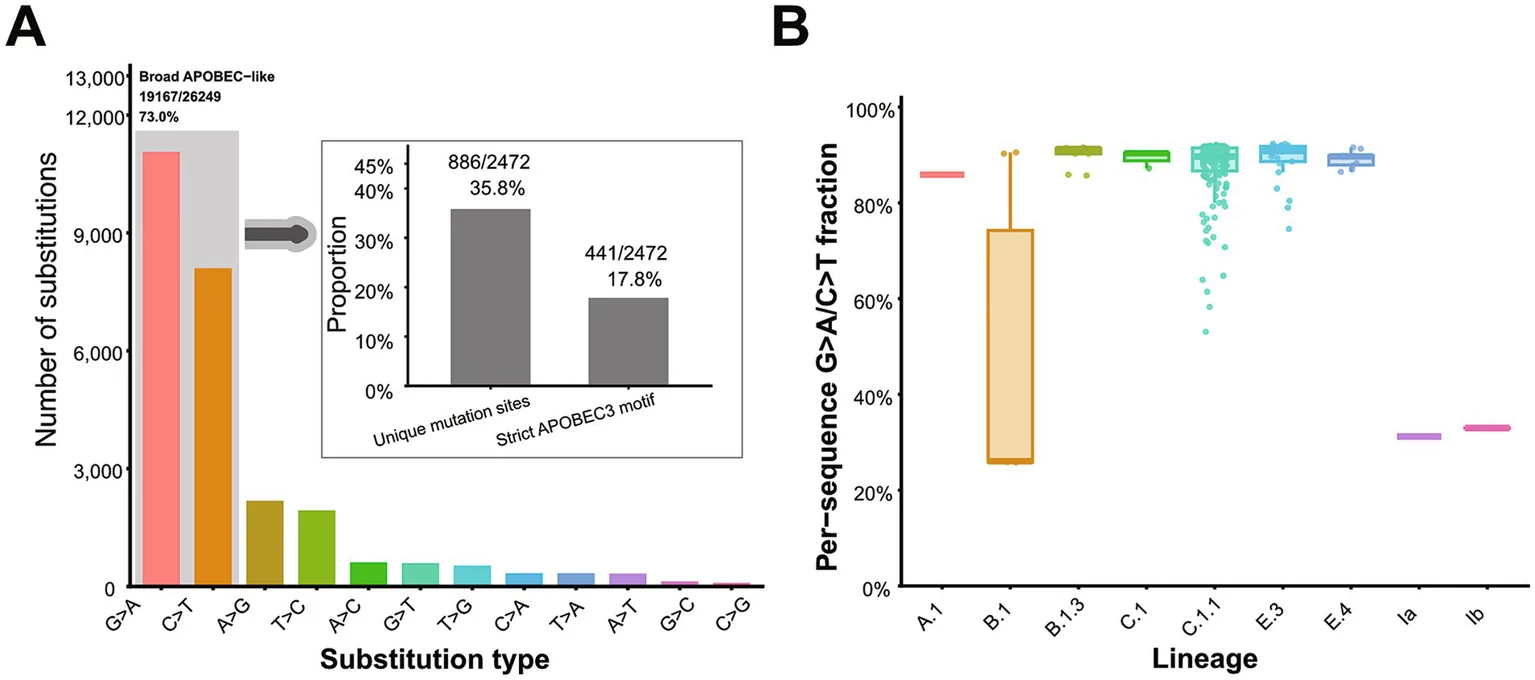
Multi-level characterization of APOBEC-like mutational signatures and their heterogeneity across MPXV lineages in Mainland China. **(A)** Mutational spectrum and multi-level characterization of APOBEC-like signatures in MPXV sequences from Mainland China. The overall nucleotide substitution spectrum shows a predominance of G → A and C → T transitions among all detected mutations. The inset summarizes APOBEC-like mutation proportions across analytical levels, when recurrent mutations were collapsed into unique genomic sites. This progressive reduction indicates that a substantial fraction of APOBEC-like mutations is shared across sequences rather than independently arising at the same genomic positions. **(B)** Per-sequence distribution of APOBEC-like transition burden across MPXV lineages. Each point represents an individual genome, and boxplots summarize the distribution within each lineage. Substantial variability is observed both within and between lineages, indicating heterogeneity in APOBEC-like mutation burden at the sequence level.

Analysis of nucleotide substitution patterns based on 230 sequences from mainland China revealed a pronounced predominance of G>A and C>T transitions, accounting for 73.0% (19,167/26,249) of all detected substitutions (Figure 5A; Supplementary Table S6). These transition types were grouped as broadly defined APOBEC-like transition classes.

To further examine the origin of this mutational bias, substitutions were analyzed at multiple levels. When collapsing mutations to unique genomic sites, the proportion of G>A/C>T transitions decreased to 35.8% (886/2,472). Under a stricter APOBEC3 motif definition, the proportion further decreased to 17.8% (441/2,472) (Figure 5A, inset). These findings indicate that a substantial fraction of APOBEC-like substitutions represents shared lineage-associated substitutions across multiple genomes rather than independently arising mutations at the same genomic positions.

At the individual genome level, the proportion of G>A/C>T substitutions varied across lineages (Figure 5B; Supplementary Table S6). Some lineages showed consistently high APOBEC-like mutation burdens, whereas others exhibited broader variability. This pattern suggests heterogeneity in mutation accumulation across circulating MPXV lineages.

## 4 Discussion

The global mpox outbreak since 2022 has been characterized by rapid international spread, evolving transmission patterns, and increasing availability of both clinical and genomic data (Alakunle et al., 2024; Callaby et al., 2025; Otieno et al., 2025; Titanji et al., 2024). In mainland China, reported cases have transitioned from early importation-associated events to locally sustained transmission clusters, accompanied by growing but uneven research output. However, a systematic understanding of how epidemiological and genomic evidence align remains limited. This scoping review integrates the available epidemiological and genomic evidence to describe transmission, lineage, and mutation patterns represented in the current evidence base, while delineating important gaps in data coverage and clinical–genomic linkage in mainland China.

At the epidemiological level, the profile of reported cases in mainland China is broadly consistent with observations from other regions affected during the global outbreak. Cases were predominantly reported among young adult men, with a high proportion of MSM exposure and frequent HIV co-infection (Sharif and Dey, 2023). Among hospital-based series reporting this variable, overseas travel history was recorded in 18/490 cases (3.7%; 95% CI 2.3–5.7%). This measure was derived from a limited subset of reported cases and did not systematically capture travel destinations, locations of exposure, or epidemiological links to early or putative index cases; it therefore cannot be used to identify the geographic source(s), number, direction, or timing of MPXV introduction events. The infrequent reporting of overseas travel is compatible with an important role of local transmission, although incomplete travel, exposure-location, and contact-tracing data preclude attribution of individual cases or lineages to imported or local transmission. This interpretation is consistent with observations from mpox outbreaks in other settings, in which limited importation-related events were followed by community-level transmission (Callaby et al., 2025; Lucero-Obusan et al., 2024). The first detected case in mainland China was reported as imported in 2022, and selected later importation-related events, including clade Ib-associated cases, have also been documented (Li et al., 2022; Song et al., 2025; Sun et al., 2025); however, the available evidence does not permit attribution of individual lineages or the overall outbreak to specific external geographic sources.

Despite the widespread occurrence of typical symptoms such as rash and fever, severe outcomes were relatively uncommon. This finding aligns with previous observations that clade IIb-associated outbreaks are generally associated with lower mortality compared with historical clade I outbreaks (Andrei and Snoeck, 2023). However, heterogeneity in hospitalization and outcome reporting across studies indicates that clinical severity should be interpreted cautiously, particularly in the absence of standardized reporting frameworks.

Together with findings from published regional genomic studies, the lineage distribution observed in the curated public dataset is consistent with the presence of multiple clade IIb lineages and heterogeneous local amplification in mainland China, rather than a single lineage expansion. The relatively frequent representation of C.1.1 and C.1 in the available public genomes should be interpreted in light of temporal and regional composition of sequence deposition and does not provide a population-representative estimate of lineage prevalence (Cao et al., 2025; Shi C. et al., 2025; Zhang et al., 2024). Likewise, the lower representation of B.1.3 (Choi et al., 2024; Otieno et al., 2025), E.3 and E.4 (Li et al., 2025; Shi C. et al., 2025; Zhao et al., 2026) reflects the diversity captured by the curated dataset and cannot establish their relative circulation at the national level. Sporadic Ib detections were associated predominantly with importation-related events (Song et al., 2025; Sun et al., 2025). Thus, the present analysis does not independently reconstruct the number, geographic sources, direction, or timing of introduction events; rather, it provides a descriptive mainland China–level synthesis that is consistent with the heterogeneous lineage circulation reported by regional genomic studies.

At the molecular level, amino acid variation provides an additional descriptive layer of genomic diversity in the curated public dataset. C.1.1 and E.3 showed relatively limited amino acid variation, whereas E.4 contained a set of substitutions distributed across multiple viral proteins. These observations describe lineage-associated patterns in the available sequences; because lineage sample sizes were imbalanced and no formal between-lineage frequency comparison was performed, they should not be interpreted as evidence of differential adaptation, fitness, or functional effects. Although some affected proteins have been implicated in viral replication or host interaction (Molteni et al., 2023), the present analysis does not establish the functional significance of individual substitutions. Experimental studies will be required to determine whether any of these changes have biological consequences.

Nucleotide substitution analysis showed a predominance of G>A and C>T transitions, a pattern compatible with APOBEC-like mutation signatures previously described in clade IIb MPXV genomes (De Pascali et al., 2025; Gigante et al., 2022; Molteni et al., 2023; Zhang et al., 2024). In the present descriptive analysis, the proportion of APOBEC-like substitutions decreased substantially after mutations were collapsed to unique genomic sites. This pattern may reflect the contribution of shared lineage-associated substitutions that were inherited and amplified through transmission chains, rather than recurrent independent mutational events alone, consistent with observations from genomic epidemiological studies (Otieno et al., 2025; Pekar et al., 2025). However, sequence-context analysis cannot distinguish the relative contributions of APOBEC-mediated editing, phylogenetic inheritance, and transmission-linked amplification. Accordingly, the observed APOBEC-like spectrum should not be interpreted as direct experimental evidence of ongoing APOBEC activity or as evidence of a causal role in viral evolution.

Beyond the descriptive genomic observations, a principal contribution of this study is its mainland China–level mapping of the structure and limitations of the available evidence. Integration of epidemiological and genomic data at the study level identified substantial heterogeneity in evidence coverage and several critical gaps. First, genomic evidence was highly concentrated in a limited number of regions, suggesting uneven sequencing capacity and potential underrepresentation of viral diversity. Second, the proportion of cases subjected to sequencing varied widely across studies, limiting comparability across datasets. Third, and most importantly, the absence of individual-level linkage between clinical and genomic data prevents direct integration of these domains, constraining the ability to assess lineage-specific differences in transmission or clinical outcomes. These findings have important implications for future research and public health practice. The observed epidemiological profile underscores the importance of targeted interventions in high-risk populations, particularly MSM communities. Future surveillance should prioritize broader genomic coverage and near-real-time linkage of sequencing, epidemiological, and clinical data.

## 5 Limitations

This study has several limitations. First, analyses were conducted at the study level without individual-level data, limiting assessment of associations between viral lineages and clinical characteristics. Aggregated numerators and denominators may include partially overlapping source populations and are clustered within studies. Accordingly, the Wilson 95% CIs describe precision conditional on the displayed counts but do not account for between-study heterogeneity, clustering, or population overlap and are not meta-analytic or population-representative intervals. Second, the literature-based design may introduce selection bias, particularly in the geographic representation of genomic data. Third, heterogeneity in study design and reporting reduces comparability across studies. Fourth, lack of standardized definitions for some clinical variables may affect aggregated estimates. In addition, mutation and genomic analyses relied on publicly available sequences without individual-level epidemiological linkage, including travel and exposure-location data, which may incompletely represent circulating viral diversity and limits distinction between within-host mutational processes and transmission-driven accumulation. Inference regarding the regional emergence and circulation of lineages should be interpreted cautiously because geographic metadata were incomplete and publicly available sequences were unevenly sampled across regions and time periods. These data-related limitations highlight the need for more standardized and integrated reporting in future mpox surveillance. Despite these limitations, this study provides an integrated synthesis of the available evidence and identifies key gaps in mpox research in mainland China.

## 6 Conclusion

This scoping review provides an integrated synthesis of the available clinical, epidemiological and genomic evidence on mpox in mainland China during the global outbreak period. The available evidence suggests that, following early importation-related events, local transmission became increasingly documented, alongside growing but geographically uneven research output. The available genomic evidence is consistent with the presence of multiple lineages and heterogeneous transmission patterns. The observed mutation spectrum is compatible with contributions from lineage-associated inheritance and APOBEC-like processes, but does not establish their relative biological contributions. Importantly, this study highlights substantial structural gaps in the current evidence base, particularly the lack of integrated clinical-genomic datasets, uneven sequencing capacity, and inconsistent reporting standards. These limitations constrain the ability to fully interpret transmission dynamics and lineage-specific characteristics. Strengthening integrated genomic surveillance and data linkage frameworks will be essential for understanding MPXV transmission and evolution and for improving preparedness against future emerging infectious disease outbreaks.
