# Supplementary material for: Clinical, epidemiological, and genomic evidence on mpox in mainland China, 2022–2025: a scoping review
Source: Front Microbiol. 2026 Aug 24;17:1880110. doi: 10.3389/fmicb.2026.1880110 (PMC13549128; doi:10.3389/fmicb.2026.1880110)
Supplement: Supplementary file 1 [file Data_Sheet_1.zip › Supplementary_Materials/Supplementary_Table_S1_Search_strategies .docx]

Supplementary Table S1. Search strategies and data sources used for literature and genomic data retrieval

| **Data source** | **Search strategy / criteria** |
| --- | --- |
| PubMed | ("Mpox"[Title/Abstract] OR "Monkeypox"[Title/Abstract] OR "Monkeypoxvirus"[Title/Abstract] OR "MPXV"[Title/Abstract] ) AND (China[Title/Abstract] OR "mainland China"[Title/Abstract] OR "Chinese mainland"[Title/Abstract]) AND (case*[Title/Abstract] OR patient*[Title/Abstract] OR clinical[Title/Abstract]OR epidemiolog*[Title/Abstract] OR surveillance[Title/Abstract]) NOT (review[Publication Type] OR editorial[Publication Type] OR comment[Publication Type] ) |
| CNKI | keywords: "猴痘" OR "Mpox" OR "猴痘病毒") AND ( (keywords: "临床特征" OR "临床表现" OR "症状" OR "皮疹" OR "淋巴结肿大" OR "发热") OR (keywords: "基因组" OR "基因特征" OR "全基因组" OR "测序" OR "变异" OR "进化" OR "突变") ) AND (year: 2022 OR 2026) AND language: Chinese. |
| Wanfang |  |
| GenBank/Genbase/GISAID | (monkeypox virus OR mpox OR MPXV) AND China AND complete genome |

NOTE:
- CNKI: China National Knowledge Infrastructure.

- Searches were conducted without date restriction up to March 2, 2026.

- Search strategies were adapted to database-specific syntax.
